# “Save Antibiotics, Save lives”: an Indian success story of infection control through persuasive diplomacy

**DOI:** 10.1186/2047-2994-1-29

**Published:** 2012-08-29

**Authors:** A Ghafur, V Nagvekar, S Thilakavathy, K Chandra, R Gopalakrishnan, PR Vidyalakshmi

**Affiliations:** 1 Consultant in Infectious Diseases and Clinical Mycology Apollo Specialty Hospital, 320 Anna Salai, Chennai, India; 2 Registrar in Infectious Diseases Apollo Specialty Hospital, 320 Anna Salai, Chennai, India; 3 Infection Control Nurse Apollo Specialty Hospital, 320 Anna Salai, Chennai, India; 4 Junior Consultant in Infection Control Apollo Specialty Hospital, 320 Anna Salai, Chennai, India; 5 Consultant in Infectious Diseases Apollo Specialty Hospital, 320 Anna Salai, Chennai, India; 6 Registrar IN Infectious Diseases Apollo Speciality Hospital, 320 Anna Salai, Chennai, India

**Keywords:** Carbapenem resistance, Superbug, Antibiotic usage, Antibiotic stewardship, Success story, Indian hospitals, Oncology, Infection control

## Abstract

**Background:**

Carbapenem resistant *Enterobacteriaceae* is a worldwide threat, with increasing prevalence in many countries. Restricted usage of higher end antibiotics, especially carbapenem is of great importance in tackling these super bugs. Purpose of this retrospective study was to analyse the impact of antibiotic stewardship activities on the prevalence of carbapenem resistant *Enterobacteriaceae* in our hospital.

**Findings:**

In the first Quarter of 2009, average usage of carbapenem group of antibiotics was 955 vials a month while in 2010, the usage dropped to 745 vials per month. Carbapenem resistant *E.coli* rate dropped from 3.7% in 2009 to 1.6% in 2010 and Klebsiella rate reduced from 6% in 2009 to 3.6% in 2010.

**Conclusions:**

Strict antibiotic stewardship strategies in conjunction with good infection control practices are useful in restricting higher end antibiotic usage and reducing the prevalence of carbapenem resistant *Enterobacteriaceae.*

## Background

Antimicrobial resistance is a significant global challenge which every hospital is trying to tackle. “Bad Bugs, No drugs” is an issue of upmost importance which can adversely affect the health care delivery systems of all countries [1]. Apollo Speciality hospital is a tertiary care oncology centre with an active Bone marrow transplant programme. Oncology patients receiving chemotherapy are severely immunocompromised, highly vulnerable to all infections. Higher end antibiotics especially carbapenem group is widely recommended and used in oncology hospitals. At the same time, resistance to this group of drugs is increasing in all countries [2-5]. Restriction of higher end antibiotics especially carbapenem group needs determination and whole hearted cooperation of all health care professionals involved in the care of these immunocompromised patients [6-9].

## Materials and Methods

**Figure 1:**
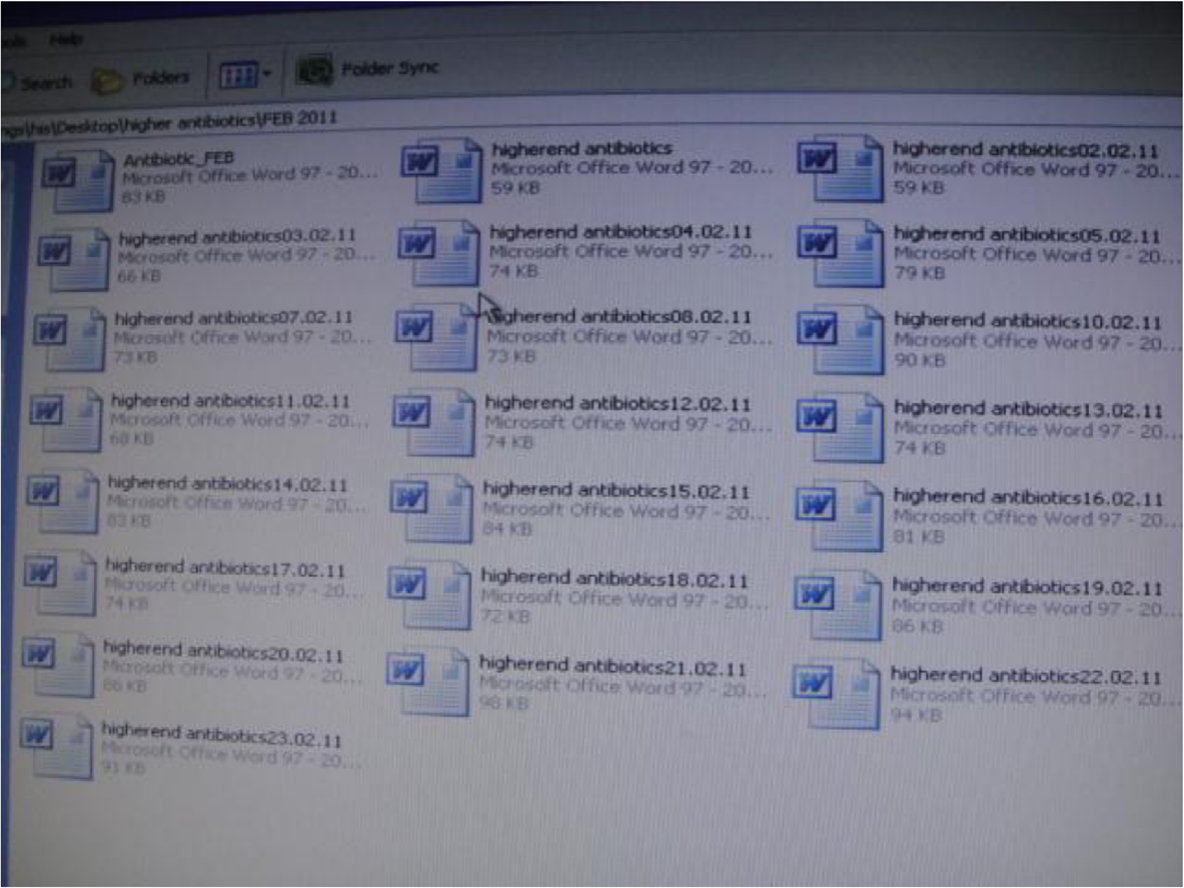
Higher end antibiotics daily check list digitally recorded.

**Figure 2:**
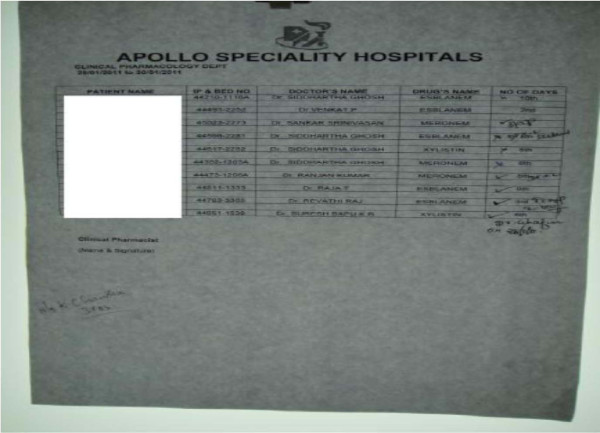
Example of a daily check list of higher end antibiotics.

**Figure 3:**
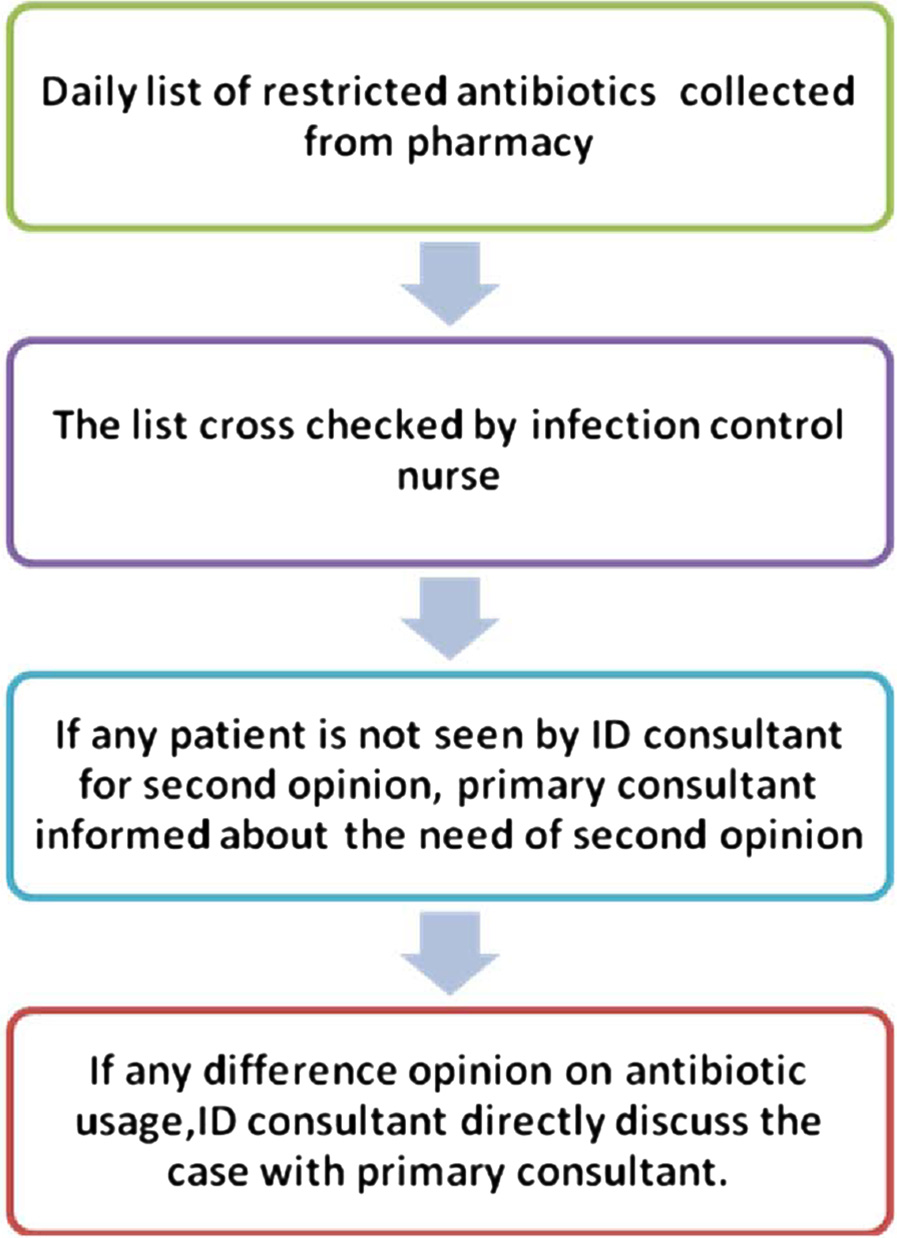
Higher end antibiotic usage tracking process.

Restricted list of higher end antibiotics was prepared (imipenem, meropenem, ertapenem, doripenem, colistin and tigecyline) by Infection Control Team in 2007. The need for a restricted list was discussed with all consultants and junior doctors by multiple circulars and academic meetings in the hospital involving all specialities. The importance of antibiotic stewardship and infection control was stressed upon in departmental meetings and so whole hearted cooperation from all medical and paramedical staff was ensured. Regular feed backs on the resistance statistics and antibiotic usage were provided to all departments. Whenever a restricted higher end antibiotic was used, second opinion by Infectious diseases Consultants within 48 hrs was made mandatory. Clinical pharmacists provided daily list of restricted antibiotics and compliance was tracked by the Infection Control Team. In case of any violation of the policy, the primary consultant was directly contacted by the Infection Control Team and if necessary by Medical Superintendent and documented. The policy was further strengthened in 2008 by regular education and person to person communication between infection control team and other doctors. Infectious disease consultant directly communicated in a diplomatic and professional way with other consultants; in case of any difference of opinion on the need of higher end antibiotic usage (Figure 1, 2, 3). Strict antibiotic stewardship policy of “Save antibiotics, Save lives” was implemented with re enforcement of basic infection control measures.

 (a) Isolation precautions were strictly followed. Sign boards for patients requiring contact isolation either for an MDR colonisation or infection were displayed on the doors of all rooms in three languages. A cart comprising of aprons, masks and gloves were placed outside the patients’ room and the usage of this was also strictly monitored.

 (b) Compliance to hand washing was regularly monitored. Infection control nurse would conduct weekly audits on adherence to hand hygiene and would give feedback to all relevant departments.

 (c) Continuing Medical Education activities were conducted periodically for all junior doctors, healthcare workers and nurses with detailed discussions on all aspects of infection control.

 (d) Education of relatives of patients: leaflets on the importance of infection control measures were regularly distributed among patients and relatives to ease any doubts on the need of such strict infection control measures.

 (e) Whenever a specific infection control measure was implemented, for e.g. Isolating in a single room, both patient and relatives were properly counseled on the need of such special measures to protect the patient and other patients in the hospital.

 (f) Infection prevention week was conducted during which our work was reviewed, detailed plan for the future programmes and activities were made and they were strictly adhered to. During this week health care workers and nurses were also awarded as a token of recognition of their efforts and also to encourage them.

 (g) Infection control committee meetings were held once in three months, to analyse the resistance trend, compliance to infection control policy and to plan future activities. The committee included representatives of all departments, hospital management, housekeeping, nursing department, infection control nurses, quality system, laundry manager and hospital engineer.

Infection control in the ICU, especially in a hospital which caters to the need of immunocompromised host and trauma patient is a great challenge. A dedicated ICU nurse solely taking care of Infection control was assigned. This nurse would monitor adherence to the Infection control practices in the ICU especially compliance to the antibiotic policy and various bundle approaches.

Adherence to surgical prophylaxis was the next challenge requiring a multidisciplinary approach. A dedicated nurse assigned for this purpose would look into the timing and choice of the antimicrobial agent, the number of doses administered and would constantly give a feed back to the surgeons and management.

## Results and Discussion

Due to very vigilant monitoring on the prescription of higher end antibiotics there was an obvious drop in the carbapenem usage. In the first Quarter of 2009, average usage of carbapenem group of antibiotics was 955 vials (imipenem 265, meropenem 645, ertapenem 43vials) a month while in 2010, carbapenem usage dropped to 745 vials (imipenem 185, meropenem 518, ertapenem 42). Antibiotic usage data available as vials used per month, DDD(Defined Daily Dose),as it ideally should have been; still serves the purpose, albeit a crude one, to compare usage over time; in a hospital where there was no difference in the bed occupancy over time, especially when the purpose of the study was not a direct comparison between carbapenem usage and carbapenem resistance, instead assessing the performance of a number of interrelated infection control practices. Carbapenem resistant *E.coli* rate dropped from 3.7%(of the total number of *E.coli* isolates in all specimens) in 2009 to 1.6% in 2010 and carbapenem resistant *Klebsiella* rate reduced from 6% in 2009 to 3.6% in 2010. Extended Spectrum Beta-lactamases producing *E.coli* rate increased from 70% to 82%,while *Klebsiella* rate reduced from 80 to 75%. As a result of the strict antibiotic policy, the adherence to the receipt of first dose of antibiotic was greater than 95% and adherence to discontinuation of antibiotic post operatively was 80%.

The reduction in the prevalence of carbapenem resistant *Enterobacteriaceae* may not be just due to the tightness of the antibiotic policy but to the improvement in the overall standard of infection control, as a result of better awareness on the importance of controlling superbugs. This policy has helped in keeping the carbapenem resistant *Enterobacteriaceae* rate lower than the previous year; at a time when increasing resistance of these bacteria to carbapenem has been reported from around the world; prompting World Health Organisation to declare antimicrobial resistance the most important challenge medical field is facing today [10].

The study did not intend to analyse the effect of any single infection control intervention, but does prove that strict infection control strategies could be effectively implemented in hospitals in developing countries. Success of our campaign has significant implication at national and international level. All hospitals in South Asia should follow similar tight antibiotic policy and infection control measures so that the region can make significant contribution to the global fight against antimicrobial resistance. Success of infection control strategies in a few hospitals will only have limited impact in the overall antimicrobial resistance rate in a country, unless similar strategies are adopted by all hospitals.

## Competing interests

AG has received lecture fees or advisory fees from multiple pharmaceutical companies. RGK has received lecture fees or advisory fees from multiple pharmaceutical companies. TS, CK, VN, PRV nothing to declare.

## Authors’ contributions

AG the primary author prepared the manuscript and played the key role in implementing the stewardship policy.VN, PRV contributed in data collection.CK contributed in implementation of the policy and data collection. TS contributed in implementation of the stewardship policy and data collection. RGK made significant contribution in initiating the antibiotic policy and assisted in writing the article. All authors read and approved the final manuscript.

## Authors’ information

AG and RGK are infectious diseases and infection control consultants in Apollo specialty Hospital.VN, PRV is a fellow in infectious diseases. TS is junior consultant in infection control.CK is infection control nurse in the hospital.
